# The Association’s Bill

**Published:** 1897-03

**Authors:** 


THE ASSOCIATION'S BILL.—Of course our readers will want to hear from the Association's bill to regulate the practice, now before the legislature. They will doubtless be surprised and disappointed to learn that, up to this writing, March 1st, the bill has made no progress, whatever, beyond being reported back to the House, as amended (at the suggestion of the homeopath's committee, who met with that of the State Association before the legislative committee), with the recommendation that it pass. The amendment referred to consists of reducing the number of members of the State board of medical examiners from twelve to eight, of whom four shall be *physicians* ["regulars"], two homeopaths and two eclectics, the homeopaths saying they will be "satisfied" with that proportion. Well, I should remark. Satisfied! Yes; they have *forced* recognition by the "despised regulars," out generalled, out manœuvered them, and have at last brought them to their own terms! The *Texas Medical News* says, "it was an occasion for rejoicing, and there was rejoicing, not loud but deep." We fail to appreciate the occasion as one for rejoicing on the part of the medical profession, and rather regard it as one more suitable for sack cloth and ashes; an occasion on which the medical profession extends full recognition as equals to a handful of the most flatant frauds and pretenders in the State—thereby sacrificing its dignity, self-respect and principle.

According to an estimate made by the chairman of our legislative committee—using Polk's Directory as his guide, out of 4627 physicians in Texas, there are seventy-five homeopaths and less than one hundred eclectics. Satisfied? I should say so.

The bill has not, at this writing, been taken up in either the House or the Senate; and as the season is far advanced, it begins to look as if it will never see day light.

Notwithstanding representatives of the so-called "schools" met with the Texas State Medical Association's committee before the committee of the legislature and agreed to the bill, it is meeting with violent opposition at the hands of all the other quacks, just as we said it would do. We have repeatedly pointed out the fact that these people have boasted that they will *oppose any bill emanating from the State Medical Association.* As in former years, the irregulars are fighting the bill, using their favorite weapons—lying and misrepresentation, and crying persecution: hiring some jackleg lawyer to write articles against it, which are published in the *Austin Daily Statesman* as paid matter. The same old cry is made, of "class legislation," "monopoly," "trying to run others out," etc., and, as in former years, they seem to have enlisted the sympathy of the general run of representatives. I find, in conversation with members, a general sentiment in favor of what they call "personal rights." It is claimed that if a man wants to employ a quack to attend his family, it is his own business; if he willing to take the risk no one else should care; he claims the right to die, or have his family slain by methods of his own choice, etc., which is all rot. But, we find it extremely difficult to make them see it in that light.

The State will not permit a person to engage in any other occupation dangerous to life, without a special permit, a license, granted only after a demonstration of fitness; and we cannot see how any man can call in question the right,—nay, the duty of the State to restrict the exercise of so dangerous and grave a privilege as practicing medicine to the hands of those especially educated in its details. It it a privilege not to be lightly granted. But, as before stated, we have, so far, failed to impress these views upon the legislators, and we fear the bill, like its predecessors, is doomed to an early grave.

So—the State Assoɔiation has humbled itself to no purpose. *Vive la bagatelle!* The quack reigns triumphant still!

As we go to press we learn that the bill is just where we left it at date of our last issue, reported back favorably in the house, and there it sticks. Sorry we are not able to give our readers a more favorable report.

Personally the writer is opposd to the bill for many reasons, but if it is the voice of the majority, we have nothing to say against its passage. One of its chief defects, in our opinion, is that under its provisions no one will be permitted to be examined for license, unless he is a graduate of a school requiring three courses in three separate years. If qualification be the test (and it ought to be the sole test), and one possesses the requisite qualification, why should it matter, how, when or where he qualified? or how long it took him? And—as to the "homos," we submit,—they are physicians, or they are not physicians. If they are *physicians* and want to *practice medicine.* they should conform to the requirements of physicians; if they are not, we have nothing to do with them. I fail to see why they should be exempt from examination or be examined by their *kind*, when they essay to *practice medicine;*—homeopathy is not medicine.

But—that's a thrice-told tale. I feel humiliated at the acaction of the State Society in giving these parties recognition as physicians,—especially as the bill, like its antecedents,—is being opposed by the rank and file of the irregulars, and will very likely be defeated.

As a specimen of the tactics being used by the quacks against the medical bill, may be mentioned their published appeal to the quacks to rally to defeat it, in which it is alleged that the bill, if passed, will interfere with a person in a choice of a physician. Every legislator who has a grain of sense will see through that; they know that no law can have a retro-active effect, so as to disqualify any doctor now practicing, who has ever qualified under the existing law, or any previous law. It can only take effect from and after its passage, and will then only cut off the incompetent who wish to practice; not those, unfortunately, who are now practicing. So, it will not interfere with the utmost freedom in the choice of a doctor. Another lie nailed.

